# Process Evaluation of an Application-Based Salt Reduction Intervention in School Children and Their Families (AppSalt) in China: A Mixed-Methods Study

**DOI:** 10.3389/fpubh.2022.744881

**Published:** 2022-03-14

**Authors:** Yuewen Sun, Yuan Li, Feng J. He, Hueiming Liu, Jingwen Sun, Rong Luo, Chunlei Guo, Puhong Zhang

**Affiliations:** ^1^Nutrition and Lifestyle Department, The George Institute for Global Health at Peking University Health Science Center, Beijing, China; ^2^Faculty of Medicine, University of New South Wales, Sydney, NSW, Australia; ^3^Barts and The London School of Medicine and Dentistry, Institute of Preventive Medicine, Queen Mary University of London, London, United Kingdom; ^4^Health Systems Science Department, The George Institute for Global Health, Sydney, NSW, Australia

**Keywords:** process evaluation, salt reduction, mHealth (mobile Health), primary school, mixed method approach

## Abstract

**Background:**

Salt reduction is a cost-effective, and rather challenging public health strategy for controlling chronic diseases. The AppSalt program is a school-based multi-component mobile health (mhealth) salt reduction program designed to tackle the high salt intake in China. This mixed-methods process evaluation was conducted to investigate the implementation of this program across sites, identify factors associated with the implementation, and collect evidence to optimize the intervention design for future scale-up.

**Methods:**

Mixed methods were used sequentially to collect data regarding five process evaluation dimensions: fidelity, dose delivered, dose received, reach, and context. Quantitative data were collected during the intervention process. Participation rate of intervention activities was calculated and compared across cities. The quantitative data was used for the selection of representative intervention participants for the qualitative interviews. Qualitative data were collected in face-to-face semi-structured interviews with purposively selected students (*n* = 33), adult family members (*n* = 33), teachers (*n* = 9), heads of schools (*n* = 9), key informants from local health, and education departments (*n* = 8). Thematic analysis technique was applied to analyze the interview transcripts using NVivo. The qualitative data were triangulated with the quantitative data during the interpretation phase.

**Results:**

The total number of families recruited for the intervention was 1,124. The overall retention rate of the AppSalt program was 97%. The intervention was implemented to a high level of fidelity against the protocol. About 80% of intervention participants completed all the app-based salt reduction courses, with a significant difference across the three cities (Shijiazhuang: 95%; Luzhou: 73%; Yueyang: 64%). The smartphone app in this program was perceived as a feasible and engaging health education tool by most intervention participants and key stakeholders. Through the interviews with participants and key stakeholders, we identified some barriers to implementing this program at primary schools, including the left-behind children who usually live with their grandparents and have limited access of smartphones; perceived adverse effects of smartphones on children (e.g., eyesight damage); and overlooked health education curriculum at Chinese primary schools.

**Conclusion:**

This process evaluation demonstrated the feasibility and acceptability of using smartphone applications delivered through the education system to engage families in China to reduce excessive salt intake.

**Clinical Trial Registration:**

The AppSalt study was registered at www.chictr.org.cn, identifier: ChiCTR1800017553. The date of registration is August 3, 2018.

## Introduction

Salt reduction is one of the best-buy strategies for controlling the burden of non-communicable diseases (NCDs) (1). Many high-income countries have implemented their national salt reduction strategies (2) and achieved a significant reduction of average population salt intake, such as Finland and the United Kingdom (3, 4). However, their experience might not be suitable for the Chinese context because of the significant differences in dietary habits. For instance, most of the salt in the Chinese diet is discretionarily added by the consumers to dishes or soups during cooking, such as salt, soy sauces, and other sauces (5).

Unhealthy dietary habits typically emerge from early childhood and result in long-term health damage in adulthood (6, 7). Therefore, cultivating healthy lifestyles to prevent related diseases is essential during childhood and early adolescence (8). A randomized controlled trial conducted in Northern China demonstrated that a school-based education program (School-EduSalt) could effectively reduce the salt intake of primary school students and their family members (9). This program exemplifies a potential cost-effective salt reduction intervention model through the education system for the Chinese context (10). However, the generalizability of this intervention model is limited because of its reliance on teachers for intervention delivery and the extra workload to schools. Built on the School-EduSalt program, a further study, named AppSalt, was designed to incorporate a smartphone application to deliver salt awareness education in order to alleviate the workload of health education on school teachers (11).

To test the effectiveness of the AppSalt program, the researchers conducted a cluster randomized controlled trial (cRCT) at 3 cities (Shijiazhuang, Luzhou, Yueyang) and recruited 54 primary schools to participate in the trial (11). These 3 cities were located in the north, southwest, and central China, which were selected for their geographical locations and relevant dietary habits differences. Half of the recruited schools at each site were randomized to the intervention group and participated in the AppSalt intervention activities for 1 year. The control group continued with their usual health education curriculum. The usual health education courses for third graded students include the following topics: infectious diseases prevention, healthy lifestyles, sight protection, smoking cessation, etc. The knowledge on salt is not covered in the usual curriculum. The primary outcome for assessing the effectiveness of the trial is measured by the difference between the intervention and control groups in the change of salt intake measured by 24-h urinary sodium from baseline to the end of the trial (11). The effectiveness evaluation found that the salt intake in intervened adults decreased by 0.82 g/day (12).

Process evaluation is recommended as an integral part of complex interventions by the UK Medical Research Council (13). With the development of modern technology, mobile phones and apps have become a popular platform for public health interventions (14). However, there is a paucity of studies investigating the implementation process of these mobile health(mHealth) interventions, participants' satisfaction with these apps, or factors affecting the implementation. Additionally, most of these mhealth programs at schools were conducted among middle/high school students (15). Fewer programs have used apps for health education among primary school students, who require parents' assistance of using phones. Therefore, the intervention mechanism of the AppSalt program is different from other mHealth interventions.

We designed and conducted a mixed-methods process evaluation alongside the cRCT to investigate the intervention process of the AppSalt program in a real-world setting. Besides, key stakeholders' opinions on scaling up the program were collected through semi-structured interviews.

## Methods

### Intervention Design and Implementation

AppSalt program is a multi-component salt reduction platform used to deliver salt reduction knowledge and skills to primary school students and their families (11). The intervention strategies were developed following the principles of Health Belief model and Socioecological model (16, 17). In this program, a smartphone app named “AppSalt” was designed for delivering standardized health education courses and tasks to third-grade students (8–9 years old) and their parents. These courses and tasks are the core intervention strategies at the individual level. Besides, offline intervention strategies were developed to enable interpersonal and community impacts for salt reduction, including parent group meetings, class competitions, and school environment cultivation. All students in the selected intervention school, who have access to smartphones with their (grand)parents' assistance and are willing to take part, are considered eligible to participate in the AppSalt program. The details of the AppSalt program intervention components are provided in Table 1. These intervention activities should be completed in two terms (~10 months). A more detailed description of the AppSalt program and its logic model are reported in previous publications (11, 18).

**Table 1:** AppSalt program intervention components.

**Intervention components**	**Description**	**Objectives**	**Ideal frequency**	**Implementer**	**Participants involved**
**App-based intervention**					
Online health education courses	9 courses on salt reduction, each with a 10-min video, a quiz, and a practical task Module 1: The basics of salt Module 2: High-salt food in our life Module 3: Low sodium salt Module 4: Misunderstandings of reducing salt intake in life Module 5: First re-cap Module 6: How to reduce salt used in cooking? Module 7: Salt and pre-packaged food Module 8: How to reduce salt when eating out? Module 9: Second re-cap	•To provide the key messages of salt reduction to students and their families	Monthly	Central program officer	Students with (grand)parent's assistance
7-day salt intake monitoring	Use the function module embedded in the App to calculate the average salt intake amount by keeping a 7-day diet diary	•To improve participant's awareness of their salt intake amount and the gap between their current intake amount and the recommended amount to regularly monitor the change of salt intake	Quarterly	Central program officer	(Grand)parents
**Offline intervention**					
Parent group meetings	Teachers organize parent group meeting at the beginning and the end of each term with CDC staff's support	•To facilitate peer communication between the participants • To collect participants' feedback	Twice every term	Teachers with CDC staff's support	(Grand)parents and students
Competitions and awards	Teachers organize some competitions regarding salt reduction. Top 30 students at each site will be awarded with certificates and prizes	•To motivate students and parents for active participation	Four times in total	Teachers with support from central program office	Students with (grand)parents' support
Supportive environment	Headteachers regularly post posters on campus	•To cultivate supportive environment for salt reduction on campus	Once every month	School principals	Publicly available to all students, teachers, and parents in the intervention schools

The implementation of the AppSalt program in the trial was facilitated by a joint partnership of multiple stakeholders, including the intervention schools, the local CDC office, local education authority, and the central program office. In this AppSalt program, the parents and students were instructed by teachers to install the App and use it for salt reduction courses and salt intake monitoring. During the implementation, the staff of the intervention schools, especially the teachers, were responsible for checking children's homework, communicating with students and parents, organizing offline activities, and making school-level motivation schemes for students. Local CDC office and education departments were responsible for providing professional support to schools and facilitating the implementation of offline intervention activities. The central program office provided technical assistance and support to partners, managed the delivery of online health education sessions, and monitored the implementation progress.

When the intervention group received AppSalt program, the control group continued their usual health education curriculum. The usual health education courses for third graded students include the following topics: infectious diseases prevention, healthy lifestyles, sight protection, smoking cessation, etc. The knowledge on salt was not covered in the usual curriculum.

### Study Design and Participants

This study was informed by the process evaluation framework proposed by Steckler et al. (19). A mixed-methods approach was used to systematically evaluate the implementation process of the AppSalt program, including fidelity, reach, dose delivered, dose received, and context. We only evaluated the intervention group in the trial. The control group was not evaluated as they continued with their usual health education and did not receive any extra intervention activities or materials from the program. The detailed demograhic characteristics of the participants in the AppSalt program could be found in our previous publication (12).

### Data Collection

#### Quantitative Data Collection

The quantitative data, including app logs, activity logs, and routine monitoring data, were collected alongside the intervention process. App usage log was generated when the users logged onto the App and used it for health education and other functions. Activity logs were kept by the teachers, which recorded the details of offline intervention activities, including the date, number of participants, and description of the activities. Routine monitoring data was collected by a program coordinator every week in the intervention process to evaluate the implementation of all intervention strategies. Preliminary analysis of routine monitoring data was conducted to identify implementation deviations and optimize the intervention process.

#### Qualitative Data Collection

The qualitative data was collected using one-on-one semi-structured interviews from October 2019 to December 2019, i.e., within 1 month after completing the 1-year intervention of the AppSalt program. In total, 32 students, 32 family members, 9 teachers, 9 heads of schools, and 8 representatives from local health and education departments were interviewed by trained researchers.

Maximum variation sampling was applied for selecting participants for the interviews (20). At each study site, we purposively selected three intervention schools to represent different implementation performance levels. From each selected school, the trained interviewers conveniently selected 3 or 4 students and their adult family members to participate in the interviews regarding their experience of participating in the AppSalt program. In addition, the interviewers also invited the teachers, and school head-teachers for the interviews to ask their experience of organizing and implementing this program. At each study site, we also invited representatives from local health and education authorities for interviews. More details are published in the protocol of this study (21).

The interviews with selected students, family members, teachers, and school principals were performed one-on-one in the classroom or office at intervention schools; interviews with local health and education authorities were performed at their office. Each interviewee was offered a gift as a token of appreciation for participating in the interviews.

### Data Analysis and Interpretation

#### Quantitative Data Analysis

The participation rate of online health education courses and offline activities were analyzed to represent participants' adherence to the intervention program. The participation rate was compared across sites. Descriptive statistics (comparisons of means, medians, or percentage as appropriate) was used to assess the implementation rates of the five intervention components of the AppSalt program.

#### Qualitative Data Analysis

Deductive analysis of semi-structured interviews was performed using thematic analysis and managed using QSR Nvivo 12 (22). The lead author (YS), a public health researcher responsible for routine monitoring of the AppSalt program, developed the preliminary coding scheme based on the semi-structured interview guides and the process evaluation framework adopted in this study. Another public health researcher (JS) independently used the preliminary coding scheme and coded 5 interview transcripts to examine the feasibility and accuracy of this coding scheme. Discrepancies emerged during this phase were discussed. We refined the coding scheme through consensus-building. Once the coding scheme was agreed by both authors, YS continued coding all the interview transcripts. The coding scheme could be found in Additional File 1. After completing the coding, researchers summarized the patterns of the codes and identified recurrent themes from these interviews. The transcripts are in Chinese, and the selected quotations were translated into English for use in this manuscript.

#### Integration

The qualitative data is triangulated with the quantitative data during the interpretation phase to explain the 5 process evaluation dimensions. The quantitative data evaluate how the implementation of the AppSalt program is achieved in different settings. The qualitative data provide complementary information to the 5 evaluation components, which allow researchers to investigate the in-depth reasons behind the varied implementation performance (23). As the fidelity and dose delivered are closely related, we combined these two components when interpretating and presenting the results of this study (24).

## Results

### Reach

The total number of families recruited for the intervention was 1,124 (Shijiazhuang: 467; Luzhou: 324; Yueyang: 333). The number of students recruited from each intervention class varied from 11 to 57, with a median of 46. This program's reach was highest in Shijiazhuang, where 96% of eligible students participated in this program. The other two sites both recruited about 72% of eligible students for participation.

In the interviews with participants, we found different initial motivations for joining this program. 19 out of 32 parents said they volunteered to participate in the program as they wanted to learn some knowledge on salt and its health impacts. About 40% of the parents said they were selected by the teachers and agreed to participate in helping the teachers complete their tasks.

Although some participants were probably not self-motivated when first joining the program, most of them completed their 1-year participation in the AppSalt program, which helped achieve a high retention rate. In total, 34 students dropped out of the program during the intervention (Shijiazhuang 7; Luzhou 13; Yueyang 14), resulting in a retention rate of 97%. These dropping-outs were mostly due to inevitable reasons, including transferring to another school or inaccessibility of smartphones happened during the intervention process.

### Fidelity and Dose Delivered

Salt reduction knowledge and skills were delivered to the participants through multiple channels in the AppSalt program, including online health education courses and tasks, 7-day salt intake monitoring, offline salt reduction activities, communication leaflets, and brochures.

#### App-Based Intervention Strategies

During the 1-year intervention period, online salt reduction courses and tasks were posted on the App following the intervention schedule. Nine online salt reduction courses, six online practical sessions, and four 7-day salt intake monitoring tasks were delivered through the App as scheduled. Each online session was open for the users for 2 weeks, from the day it was posted.

#### Adaptation Made During the Intervention

The central program office performed weekly supervision of app usage and implementation status to ensure timely monitoring of program implementation status and identifying potential implementation deviation. After about 4-months of implementation, the researchers noticed that many users answered the after-class quizzes without watching the health education videos. This issue would reduce the amount of knowledge received by the participants. To improve the dose of intervention received, the researchers adapted the App, which made watching 80% of online videos a mandate pre-requisite for answering the quizzes.

#### Offline Intervention Strategies

The fidelity of implementing offline intervention activities varied across schools in the three study sites, especially for the parent group meetings. Four class-level activities were designed in the protocol and implemented in all intervention schools by teachers during the intervention. The fidelity of implementing offline activities was higher in Shijiazhuang than that at Luzhou and Yueyang.

**Figure 1:**
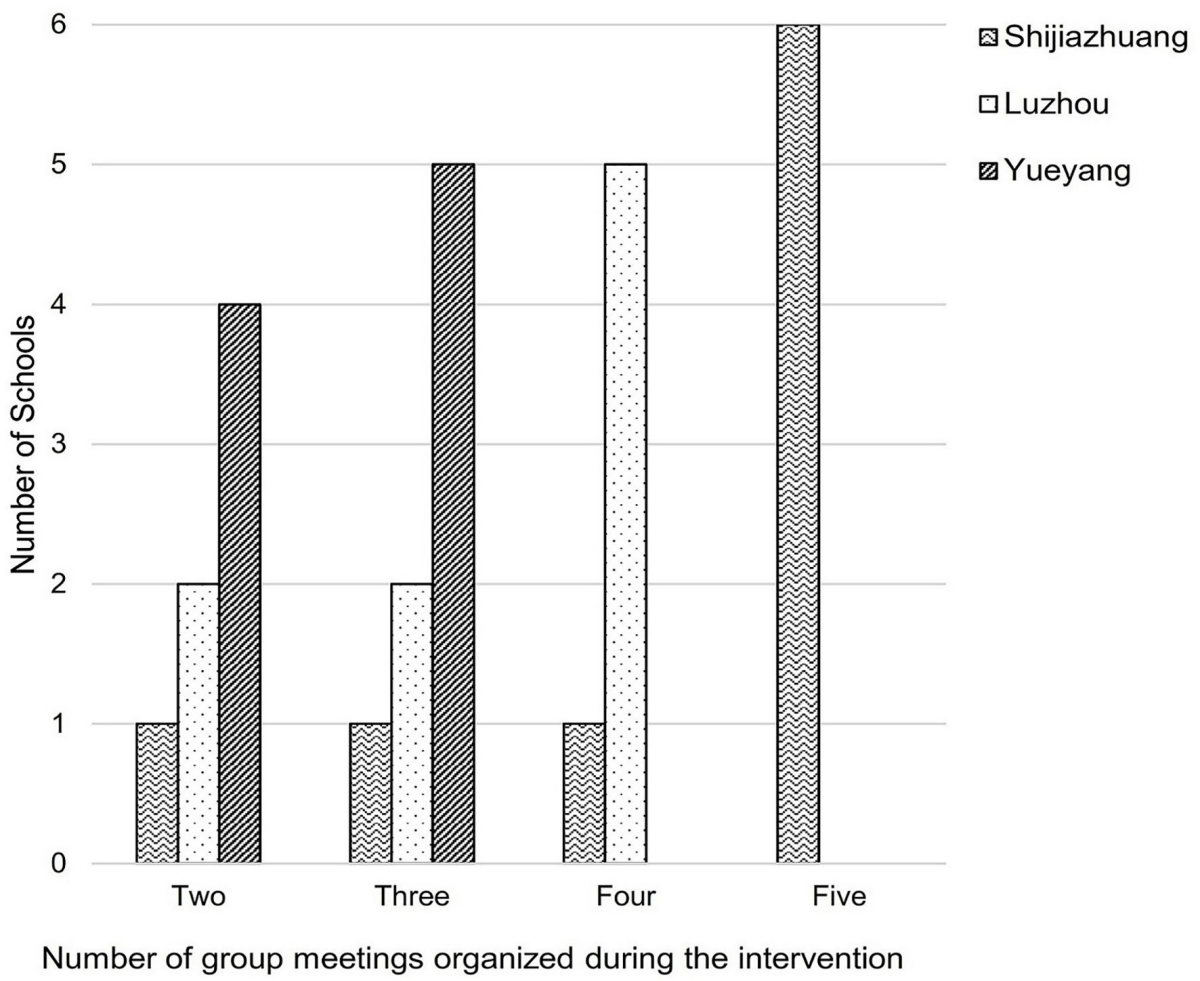
Number of parent group meetings held at intervention schools.

Four parent group meetings were required in the protocol and should be delivered at the beginning and the end of both terms. Of the 27 intervention schools, 12 schools completed four or more group meetings, eight schools did three group meetings, and the rest seven schools did only two group meetings (Figure 1). All intervention schools in Yueyang did not manage to deliver the required number of parent group meetings.

#### Factors Affecting the Implementation of Offline Intervention Activities

Teachers and school staff were the focal implementers of this program and should implement the offline activities with support from local CDC staff. Their experiences and perceptions of the salt reduction intervention would cause a remarkable difference in the intervention dose delivered to the participants, especially for those offline intervention activities. Almost all teachers recognized the importance of reducing salt intake and said they learned some salt reduction knowledge from this program.

One teacher said

“*In the beginning, I considered this program as a task from the city education department. Then I gradually realized the importance and benefits of salt reduction. Now, I would not eat any high-salt food, and even do not want to look at these foods.” (Teacher A, School A, Luzhou)*

However, many teachers said that this program created extra time demands on their already-tight schedule, which made them feel burned out. Therefore, they could not fully implement this program as required, especially the parent group meetings. Some teachers mentioned that the biggest challenge for implementing this program was communicating with students' parents and asking them to complete the tasks on time, because most parents thought this program was extra work on their schedule.

“* I have to use my spare time to work for this project, including communicating with students' parents and making plans for motivating students, which was challenging for me.” (Teacher B, School B, Luzhou)*

“* When parents approached me with a question on this program, I had to ask the CDC staff because I was not trained in that field. Luckily, the CDC staff provided much support. Then I added him to our class WeChat group so that the parents could ask him questions directly.” (Teacher A, School A, Yueyang)*

“*Communicating with students' parents was the greatest difficulty for me when delivering the program. First, this is a long-term program. Second, this program does not have much to do with my teaching. I am more used to teaching students rather than pushing parents.” (Teacher C, School C, Luzhou)*

### Dose Received

#### The Dose Received of Online Intervention Activities

Participation in online intervention strategies was high at three study sites. About 80% of participants completed all nine sessions of online salt reduction courses; about 75% of participants completed four or more times of 7-day salt reduction monitoring during the intervention process. As shown in Table 2, the online intervention strategies achieved a significantly higher participation rate in Shijiazhuang than in Luzhou or Yueyang.

**Table 2:** Participation in intervention activities at three study sites.

**Intervention strategies**	**Shijiazhuang** **(*N* = 467)**	**Luzhou** **(*N* = 324)**	**Yueyang** **(*N* = 333)**	**Total** **(*N* = 1,124)**
**Online courses**				
Completed <6 sessions	0.21%	5.56%	5.99%	3.46%
Completed 6–8 sessions	5.13%	21.06%	29.94%	17.23%
Completed all 9 sessions	94.66%	72.84%	64.07%	79.31%
**Practical tasks**				
Completed <4 tasks	1.50%	14.81%	16.77%	9.86%
Completed 4 or 5 tasks	12.39%	26.23%	38.32%	24.07%
Completed all 6 tasks	86.11%	58.95%	44.91%	66.07%
**7-day salt intake monitoring**				
0–2 times	1.7%	11.7%	14.7%	8.44%
3 times	6.0%	29.9%	17.1%	16.18%
4 times	38.2%	25.9%	35.7%	33.96%
More than 5 times	54.1%	32.4%	32.4%	41.42%
**Offline competitions**				
1st knowledge competition	98.9%	95.7%	94.6%	96.7%
2nd knowledge competitions	99.4%	91.0%	83.5%	92.2%
Art competition	83.4%	55.0%	74.2%	72.5%
Composition competitions	95.9	85.5%	75.5%	86.9%

#### The Dose Received of Offline Intervention Activities

The participation rate of offline activities varied across three sites and was highest in Shijiazhuang. The knowledge competitions were the most popular activities, while the art competition was the least popular among the participants (Table 2).

#### Satisfying Experiences of Using the App Among Participants

The participant's experience of using the App was generally satisfying, especially for those younger parents. Most parents said that the App was easy and convenient to use. In the interviews, several parents mentioned the difficulties encountered in using the App. For example, the volume of videos was low for the first several sessions; the App's installation was not easy compared with installing other apps. Most of these problems were resolved quickly by the information technology (IT) team.

#### Suggestions for Improving the Contents and Design of Intervention Materials

The videos, posters, leaflets, brochures are the main intervention materials used in the AppSalt program. Suggestions for improving the course videos consistently emerged in the interviews. Three students and 3 teachers said that the videos were a bit dull for watching and could be improved and made more interestingby bringing in cartoon characters.

Regarding the article-based intervention materials (leaflets and brochures), 22% (7/32) of the interviewed adult participants said they did not read these things because they did not have time. In addition, 50% (16/32) of the interviewed adults thought these materials were not helpful for reducing salt intake. As for the posters, 4 out of 9 interviewed teachers said that the posters could not attract much attention from the students and should be designed according to schools' context and needs.

#### Polarized Feedbacks on the Effectiveness of 7-Day Salt Intake Monitoring

Participants' feedback on 7-day salt intake monitoring was polarized. Most adult participants thought it was a useful tool to monitor the family's salt reduction progress and make them aware of the gap between their current salt intake level and the recommended amount of salt intake.

“*This (7-day salt intake monitoring) is a handy tool, which could give you a subjective amount of your salt intake. When I saw the result of the first 7-day salt intake monitoring, I realized that my family members and I ate too much salt, and we have to reduce it.” (Parent A, School A, Shijiazhuang)*

Some participants said that 7-day monitoring took too much time and effort to complete. And some participants were concerned about the accuracy of the monitoring results.

“*This task cost too much time and energy. First, we needed to weigh the take-out food and record its weight whenever we eat it. Second, it took seven days to complete. Sometimes, I might forget to fill it out and need to restart it from the beginning.” (Parent A, School B, Luzhou)*

“*I doubt the accuracy of its results. Because in the process, I was not sure if I put the right information in the right place. If the information was not accurate, I could not be sure of the accuracy of its results.” (Parent C, School C, Yueyang)*

#### Knowledge Sharing Within the Intervention Families

Knowledge sharing on salt reduction within the intervention families was bidirectional, either from children to parents/grandparents or from parents to children. Most parents said that they would encourage the students to share the salt reduction knowledge with their grandparents. Some parents said that they would personally watch the online courses first and then tell their children about salt knowledge in the online courses.

“*My mother (student's grandmother) prepares meals for the whole family, and the meals were salty before we took part in this program. After we joined this program, I started to tell them the salt reduction knowledge. And we are gradually reducing salt intake this year.” (Parent B, School B Luzhou)*

### Context

For identifying enabling and barriers factors for implementing this program, we qualitatively interviewed selected participants and key informants. From these interviews, many key stakeholders mentioned that some public health programs relevant to salt reduction were being implemented regionally, which could raise public health awareness and therefore promote the implementation of the AppSalt program at schools.

Although the raised awareness of salt intake might be beneficial for implementing the AppSalt program in primary schools, we also identified some barriers for implementation and scale-up in the interviews with selected participants and key stakeholders. The most noted barriers are as follows.

#### Perceived Adverse Effects of Using Mobile Phones on Students

Many interviewees, including 4 parents, 5 teachers, 3 school principals, and 2 representatives from local authorities, mentioned their concerns about letting children use smartphones because of the potential damage to their eyesight. Moreover, several interviewed parents said that they would limit their child's screen time per day and would not give them smartphones if it is not necessary. In addition, some interviewees were concerned that using mobile phones would make the students exposed to the temptations of games and other things. One school principal said in the interview that:

“*App has its advantages for adult users, such as convenience and flexibility. Every adult has his/her own phone and are willing to use it for self learning. However, the students usually do not have a phone for themselves. If you let them(students) to use the phone for education, they might use it for something else, such as games. Therefore, the teachers and parents can not relax at all.” (School head, School B, Yueyang)*

#### Left-Behind Children

In the interviews with teachers and school headteachers in Luzhou and Yueyang, left-behind children were widely recognized as a barrier to implementing the AppSalt program in these two sites. The left-behind students usually live with their grandparents, as their parents left the hometown for job opportunities in big cities. The accessibility to smartphones might limit the availability of the intervention content among these left-behind children. A school headteacher of a primary school in Luzhou said in the interview:

“*More than 10 students live with their grandparents. Some of these grandparents do not have a smartphone. Therefore, our participation rate is relatively low compared with other schools.” (School head, School B, Luzhou)*

#### Current Health Education Curriculum at Primary Schools

From the interviews with teachers and school headteachers, we perceived the difficulties of health education at primary schools. Most school headteachers said that they did arrange health education courses once every 2 weeks according to education authorities” requirements. However, schools usually do not have trained teachers to deliver health education courses. Therefore, these health education classes were generally delivered by the Physical Exercise (PE) teachers or the school doctors. In addition, there is no standard textbooks and evaluation of the health education delivered at primary schools.

### Stakeholders' Suggestions for Scaling up the Program

To scale up the AppSalt program in broader settings, we asked the interviewees their opinions for refining the program design for scaling up. The followings are the suggestions collected.

#### To Empower the Teachers for Delivering Health Education

In the interviews, some teachers expressed their worries for delivering health education courses to students and their parents as they lacked the expertise and professional training. During the implementation process, the teachers usually relied on the local CDC staff's support to teach the salt reduction knowledge and skills at parents group meetings. For scaling up the program, more professional training are required to empower teachers for delivering health education courses. A representative from the local education department raised her concerns for empowering teachers.

“*Health education requires professional training. For example, although salt reduction is not a very difficult topic for our teachers, they still need to prepare for this course in advance. Where should they get the knowledge? Who should provide the textbook? Who should provide the training to teachers?” (Informant A, Education department, Shijiazhuang)*

#### To Design More Effective Motivation Schemes for Teachers and Students/Parents

In the interviews with parents and teachers, some interviewees said that this program caused many extra time demands on families and teachers. To better motivate the students and parents for participation, some teachers designed their student motivation scheme. However, as for teachers themselves, there was no effective motivation scheme to implement this program. In the interviews, the teachers were asked what kind of motivation would be effective for them to implement this program. Most teachers said that the recognition from the local education department, such as certificates or awards, would be more valuable for them compared with monetary motivations.

#### To Incorporate Salt Reduction Contents Into the Regular Health Education Curriculum

Most teachers and heads of schools agreed that it might be easier for them to deliver the salt reduction courses if the content of salt reduction could be incorporated into the health education curriculum of primary schools. Almost all teachers, school heads, and representatives from local education departments agreed on the feasibility and potential beneficial effects of delivering salt reduction knowledge through a regular health education curriculum.

## Discussion

### Summary of Major Findings

Salt reduction is an urgent and challenging public health issue for China (25, 26). The AppSalt program proposed an app-based multi-component intervention program through the education system aiming to engage primary school students and their family members to reduce salt intake (11). This article systematically evaluated the implementation process of the AppSalt program in 27 primary schools to enlighten the “blackbox” between design and outcome (27). A summary of key quantitative and qualitative findings is provided in Table 3. The mixed data indicates that all online intervention activities, including health education courses and salt intake monitoring, were delivered to high fidelity against the protocol and achieved high participation rate. This study demonstrated that the smartphone app is widely accessible among the participants and could be a scalable tool for implementing health education through primary schools. Although the variation of implementing offline intervention activities was observed across the intervention sites, the overall dose delivered and participation rate reached an acceptable level at most intervention schools.

**Table 3:** Quantitative and qualitative findings for explaining the process evaluation dimensions.

**Evaluation dimensions**	**Quantitative findings**	**Qualitative findings**
Reach	Number of families recruited: • Total: 1,124; • Shijiazhuang: 467; Luzhou: 324; Yueyang: 333 Proportion of eligible participants reached: • Shijiazhuang: 96% • Luzhou:72% • Yueyang: 72% Retention rate: 97%	Motivation of participating in this program: • Wanted to receive more knowledge on salt and its health impacts • Selected by teachers and chose to cooperate Reasons for dropping out: • Transferring to other schools • In accessibility to smartphones happened during the intervention process
Fidelity and dose delivered	App-based courses and tasks: high fidelity achieved; all nine courses and tasks were delivered on schedule Class activity: moderate fidelity achieved at three sites Group meetings: varied fidelity across 3 sites; 44% of intervention schools organized enough meetings; higher fidelity achieved in Shijiazhuang Supportive environment cultivation: low fidelity Adaptation made during the intervention: watching videos was made a must for completing courses.	Teachers' experience of implementing this program: • Learned some health knowledge for themselves. • Demanding tasks of implementing this program • Had difficulties of communicating with parents regarding this program
Dose received	Average participation rate: • App-based interventions: ° Courses: ° Practical tasks: ° 7-day salt intake monitoring: • Offline intervention activity	•General satisfying experience of using the app • Improving the contents and design of intervention materials, especially those article-based materials, e.g., posters. • Polarized feedbacks regarding 7-day salt intake monitoring • Bidirectional knowledge sharing happened in the intervention awareness
Context		Facilitators: • Relevant public health policies being implemented • Raised public awareness regarding salt reduction Barriers: • Perceived adverse effects of smartphones on children • Left-behind children; • Overlooked health education curriculum

Although the implementation of the AppSalt program was generally acceptable at all three sites, higher participation rate in intervention activities and better participants' experience are observed in Shijiazhuang than the other two sites. There might be several possible socio-economic explanations for this phenomenon.

First, the intervention families in Shijiazhuang is generally better off than those in the other two sites, given the 12-month household income collected in the baseline survey (Additional File 2). Public health awareness is closely related to participants' socio-economic background, especially for the low- and middle-income countries (28). Therefore, our participants in Shijiazhuang were possibly more aware of salt's adverse effects and thus more interested in public health programs. Second, people of higher income and higher education are usually more used to using smartphones (29, 30). Therefore, an mhealth program using a smartphone app as the primary intervention tool tends to be better implemented in higher-income regions. Finally, the issue of left-behind children is more prevalent in Luzhou and Yueyang than in Shijiazhuang. The topic of left-behind children often emerged in interviews with key stakeholders in these two regions. The left-behind children usually live with their grandparents and do not have access to smartphones (31). Although the schools in Luzhou and Yueyang achieved a relatively high participation rate in intervention activities, the researchers should not overlook the issue of left-behind children when scaling up the AppSalt program.

### Comparison With Existing Studies

The rapid development of mobile technology brings tremendous opportunities for public health interventions, including smoking cessation, chronic disease self-management, weight control (32–35). mHealth apps are also considered promising tools for delivering health education among students in school settings. A systematic review finds that school-based mHealth interventions effectively reduce multiple risk factors, including unhealthy diet, physical inactivity, smoking, and alcohol use (18). Most of these programs are conducted among middle or high school students who have their own mobile phones. However, unlike other mhealth school-based interventions among adolescents, this AppSalt program relies on parental participation for achieving the intended outcome, as the third-grade primary school students were too young to use smartphones by themselves. Therefore, the implementers, especially the teachers, need to effectively motivate not only the students, but also their parents to participate in this program.

The equity issue of using the smartphone app as a primary intervention tool is also a concern that might limit the generalizability of mHealth programs (36, 37). The left-behind children usually live with their grandparents and suffer from multiple neglects compared with their peers (38). This study also indicates that the left-behind children do not have adequate access and support for using smartphone apps for online health education. The national monitoring data of migrant workers indicated that 30.1% of migrant workers left their children at home (38). This issue of left-behind children should be taken into consideration when promoting this program on a broader scale.

### Implications for Future Scaling up

This mixed-methods process evaluation systematically examined the implementation of the AppSalt program at the intervention schools, whose findings will be used to speed the translation of the AppSalt intervention to a broader scale-up (39).

Primary school health education is an inter-disciplinary task, which calls for close cooperation of schools, local health departments, and local education departments. In the AppSalt program, the rapport established between the AppSalt implementers, the teachers, and the participants is a crucial factor that facilitated the delivery of the program. For broader scaling-up, the local CDC office might not have the capacity and enough personnel to support every individual school for implementing this program. Therefore, more empowerment training should be provided for teachers and enable them to deliver health education knowledge to students and parents. Besides, standardized course materials, including textbooks, slides, and videos, should be developed and provided to schools to support the delivery of health education courses. What's more, we suggest national health education authorities to incorporate salt reduction knowledge into usual health education curriculum, which would benefit the scaling up of this program and reduce the extra burden on headteachers to deliver relevant knowledge.

Although almost all intervention schools in the AppSalt program managed to achieve an acceptable or higher participation rate, the implementation variation should be considered when promoting this program to other regions. This process evaluation indicates that the AppSalt program is generally better implemented in higher-income regions. This finding is consistent with a nutrition education program conducted at Chinese primary schools (40). For the future scale-up to other provinces in China, the researchers need to accommodate more flexibility in the intervention design for various socio-economic settings and address the barriers identified through this study. For example, a combination of online and offline salt reduction intervention packages should be designed, which would allow schools to choose suitable intervention activities according to their local context and therefore improve the scalability of this program.

### Strengths and Limitations

This study is a comprehensive process evaluation that involves mixed methods and multiple stakeholders. Both quantitative and qualitative data were used to evaluate the implementation process, and multiple stakeholders were interviewed in this study.

The study also has some limitations. First, only the participants from the intervention group are interviewed. Without evaluating the control group, we might overlook the contamination of the controlled arm during the study. However, we presume that the contamination effect should be minimal as the access to the App is strictly managed by the project office, and only the students in the intervention group were granted the account to use the App. Second, the selection of the informants from local health and education departments was carried out by local research collaborators (local CDC), which could also result in some recruitment bias. To minimize such bias, the researchers communicated in advance with local collaborators to explain the recruitment criteria. Third, although the schools were selected based on the maximum variation sampling methods, the interviewees, including parents and students, were actually self-volunteered to participate in the interviews from each selected school. We should acknowledge the possibility of recruitment bias brought by this convenience recruitment method. Fourth, we did not perform any observations to subjectively evaluate the implementation of this program in real settings due to time and personnel constraints. All monitoring data were collected with the implementer's/teacher's assistance. Therefore, some implementation problems might be neglected or not reported.

## Conclusions

School can and should become a primary setting for children to receive health education and build up healthy life habits (41, 42). However, the health education curriculum in Chinese primary schools is usually overlooked and not followed due to the burden of getting higher marks for “more important” lessons.

The AppSalt program demonstrated the feasibility of a new mHealth model for providing health knowledge by using smartphone apps. This multi-component app-based school-based salt reduction program indicated that smartphone applications are feasible tools to provide salt reduction knowledge and skills to primary school students and their family members. Further work is underway to scale up the program to larger populations in diverse settings.

## Data Availability Statement

The raw data supporting the conclusions of this article will be made available by the authors, without undue reservation.

## Ethics Statement

The studies involving human participants were reviewed and approved by Peking University Institutional Review Board (No. IRB00001052-19096) and the Queen Mary Ethics of Research Committee (QMERC2020/22). Written informed consent to participate in this study was provided by the participants' legal guardian/next of kin.

## Author Contributions

YS and RL designed this process evaluation with substantial support from the program management team and partners, especially YL, PZ, FH, and HL. CG performed qualitative data collection for this study. YS performed the data analysis and interpretation with JS's support. YS drafted the first version of this manuscript. YL, FH, HL, and PZ reviewed and revised the manuscript critically. All authors read and approved the final manuscript for publication.

## Funding

The National Institute of Health Research (NIHR) funded Action on Salt China at Queen Mary University of London using Official Development Assistance (ODA) funding (16/136/77). This study is a sub-study of Action on Salt China project.

## Conflict of Interest

FH is a member of the Consensus Action on Salt and Health (CASH) group, a non-profit charitable organization, and its international branch World Action on Salt and Health (WASH) and does not receive any financial support from CASH or WASH. The remaining authors declare that the research was conducted in the absence of any commercial or financial relationships that could be construed as a potential conflict of interest.

## Publisher's Note

All claims expressed in this article are solely those of the authors and do not necessarily represent those of their affiliated organizations, or those of the publisher, the editors and the reviewers. Any product that may be evaluated in this article, or claim that may be made by its manufacturer, is not guaranteed or endorsed by the publisher.
