# Supplementary material for: Process Evaluation of an Application-Based Salt Reduction Intervention in School Children and Their Families (AppSalt) in China: A Mixed-Methods Study
Source: Front Public Health. 2022 Mar 14;10:744881. doi: 10.3389/fpubh.2022.744881 (PMC8963959; doi:10.3389/fpubh.2022.744881)
Supplement: Supplementary file 1 [file Data_Sheet_1.docx]

**Additional file 1 Coding scheme**

**AppSalt Process Evaluation**

Coding scheme V1.0

| Code |
| --- |
| 1 Intervention strategies |
| 1 Online health education |
| App |
| Difficulties of using the tools |
| Easy to use |
| Not suitable for this family |
| Suitable for this family |
| Audience of the videos |
| Parents alone |
| Parents together with the children |
| Students alone |
| Knowledge sharing within the family |
| Salt reduction knowledge received |
| 2 Salt intake monitoring |
| Difficulty of doing 7-day salt intake monitoring |
| Easy |
| Student's involvement |
| Useful |
| Useless |
| 3 Offline Activities |
| Additional activities organized by teachers |
| Did not participate |
| Do not know the activity |
| Not interested |
| Difficulties of organizing or participating in the offline activities |
| Effects |
| Not familiar with the offline activities |
| Participation in activities |
| Like to join |
| Awards and prize |
| Interesting |
| 4 Group meetings |
| Activities and impacts |
| Difficulties of organizing group meetings |
| Frequency |
| Participation |
| 5 Supportive environment cultivation |
| Brochures |
| Did not read |
| Helpful |
| Not helpful |
| Environment building-posters |
| Did not notice |
| Helpful |
| Not helpful |
| Noticed |
| Salt reduction tools |
| Salt container |
| Spoon |
| not useful |
| unfamiliar with this tool |
| useful |
| Frequency of intervention activities |
| OK, manageable |
| Too much |
| Most useful intervention module |
| Group meeting |
| Offline activities |
| Online videos and quizzes |
| Salt intake monitoring |
| Motivation of participating |
| Health related reasons |
| Teacher's request |
| 2 Attitudes of salt reduction |
| Do not care |
| Easy to reduce salt |
| Hard to reduce |
| Not supportive |
| Supportive |
| 3 Contextual factors |
| Barriers |
| Adverse effects of mobile phones |
| Availability of mobile phones |
| Left- behind children |
| Current health education curriculum |
| Dietary habit |
| Difficulty of changing the elderly's diet |
| Local dietary preference |
| Restaurants |
| Facilitators |
| Raised awareness of salt reduction among the public |
| Salt reduction policies |
| School level support |
| Support from related government departments |
| 4 Suggestions for scaling up |
| Implementation suggestions |
| Improving school's cooperation |
| Improving the app |
| Incorporate salt reduction knowledge into regular curriculum |
| Deliver the knowledge through actual class |
| Merge the salt reduction program at schools into broader public health policies |
| Support from local government departments |
| Use student's impacts on the whole family |
| More Salt reduction skills |
| Propaganda |
| Local health and education departments |
| Difficulties encountered during the program |
| Inter-department cooperation |
| Role of local CDC in the program |
| Role of local education department in the program |
| Teachers |
| Impacts |
| Useful motivations |
| Monetary incentives |
| Recognition |
| Workload |
| Heavy |
| Manageable |

**Additional file 2 Household income and education level of intervention families in AppSalt program**

| Variable | Shijiazhuang | Luzhou | Yueyang |
| --- | --- | --- | --- |
| 12-month household income (%) | | | |
| less than 10,000 yuan | 0.9% | 4.9% | 1.9% |
| 10,001-25000 yuan | 6.3% | 7.8% | 8.9% |
| 25,001-50,000yuan | 15.9% | 24.9% | 12.1% |
| 50,001-100,000yuan | 35.6% | 34.0% | 28.1% |
| 100,001-250,000yuan | 32.9% | 23.0% | 41.9% |
| 250,001-500,000yuan | 6.0% | 2.9% | 5.1% |
| above 500,001yuan | 2.4% | 2.6% | 1.9% |
| Parental education level | | | |
| 0-6 years | 11.1% | 32.7% | 12.5% |
| 7-9 years | 24.2% | 28.4% | 23.7% |
| ≥10 years | 64.6% | 38.8% | 63.8% |
